# The Legal and Moral Responsibility of Dentists in the Administration of Nitrous Oxide Gas

**Published:** 1883-08

**Authors:** J. Allen Osmun

**Affiliations:** Newark, N. J.


### THE LEGAL AND MORAL RESPONSIBILITY OF DENTISTS IN THE ADMINISTRATION OF NITROUS OXIDE GAS.

BY DR. J. ALLEN OSMUN, NEWARK, N. J.

Read before the New Jersey State Dental Society at Asbury Park, July 18th, 1883.

*Mr. President and Gentlemen :*

I propose to occupy your attention to-day with a brief review of this important subject.

It is not my intention to go into the details of the various methods of manufacturing this agent, or the sources from which it is obtained, or its chemical constituents, or its action on the human system; these are facts which do not properly come within the scope of this paper.

Anæsthesia has been extolled to the skies by many, as the greatest boon that has ever been conferred upon the human family for the relief of suffering during painful and tedious operations, while it is quite as usual to hear it condemned in the most severe language, and by minds of no mean order. That both are absolutely correct cannot be true, for there must be well-founded reasons to lead men of intelligence to such diverse decisions. It must, therefore, be the apparent lack of judgment and knowledge of its effects upon the human economy that is shown in its administration, and of the nature of the agents used to produce this state of unconsciousness in the subject, that bring men to such different conclusions.

Dr. E. R. Squibb, in an address before the State Medical Society of New York, used the following language in referring to this subject :

"The condition of perfect anæsthesia is one of the most grave and frightful conditions of life, and by suspending more than half of vitality it comes so near to death that it is wonderful to reflect how near the boundary line can be approached, and yet so rarely passed. The issues of life and death are narrowed to a few minutes; add to this the fact that it rests with the physician whether to produce it or not, and it is difficult to understand how its importance can be over-estimated."

The above quotation is from an address delivered by a man who is, perhaps, more able to speak authoritatively than any one else his experience being of the widest extent, and I earnestly commend his remarks to your most thoughtful consideration.

That there is now and then a death from the administration of nitrous oxide is not disputed, and that being admitted it is next in order to see how far a professional man is legally responsible for his actions, and also to ascertain whether there is any difference between the legal and moral obligations, and what these differences may be.

The question of the legal responsibility of a physician, surgeon, or dentist, for unskillful treatment of a patient, is one not easily determined. There are in law no arbitrary rules by which we can fix upon the exact dividing line between absolute mal-practice and simple unskillful professional work.

In the eyes of the law, a professional man is an expert in his particular field, and he must possess a fair average amount of information in his specialty, and use his knowledge in a reasonably competent and skillful manner.

The case of Lamphier vs. Chipps, reported in the 34th volume of Common Law Reports, is in point. This was a case where the patient sued a physician for damages resulting from an alleged unskillful treatment of a bruised hand. In his charge to the jury Chief Justice Tindal stated the principles by which all cases of this character must be decided, and his remarks are so general that they would be applicable to almost any conceivable case.

The Chief Justice in summing up, after stating the pleadings, said to the jury:

"What you will have to say is this; whether you are satisfied that the injury sustained is attributable to the want of a reasonable and proper degree of care and skill in the defendant's treatment. Every person who enters into a learned profession undertakes to bring to the exercise of it a reasonable degree of skill. He does not undertake, if he is an attorney, that at all events you shall gain your case, nor does a surgeon undertake that he will perform a cure, nor does he undertake to use the highest possible degree of skill. There may be persons who have higher education and greater advantages than he has, but he undertakes to bring a fair, reasonable, and competent degree of skill, and you will say whether in this case the injury was occasioned by the want of such skill in the defendant. * * * The question is, whether this injury must be referred to the want of a proper degree of skill and care in the defendant."

Under this charge the jury gave the plaintiff one hundred pounds damages.

From the judge's charge in this case, we see that the question whether an alleged injury is attributable to a want of ordinary skill or care on the part of a practitioner, is simply a question of fact for the jury to determine.

It is well for us just here to inquire what we are to understand by ordinary skill. We recognize the fact that the grade of skill increases with, or in proportion as the delicacy and danger of the operation increases. Ordinary skill in an occulist is more than ordinary skill in a less delicate branch of surgery; and in dentistry such skill is higher as you progress towards the more complex and dangerous class of operations.

The Marine Court of New York lately rendered a judgment against a dentist for an alleged want of skill and care in the administration of nitrous oxide gas.

In this case the judge laid down a slightly stricter rule than that of some former decisions. Briefly stated, the story of the case is this: A patent applied to take gas and have a tooth extracted. The gas was administered, and the tooth was broken in such a manner that part of it dropped down the patient's throat, lodging in the bronchial tubes. It took the man a month to cough it up, during which time he suffered severely.

The court said, that as an anæsthetic deprived the patient of the control of his faculties and rendered him unable to take any precautions or make any efforts for his own safety, the physician or surgeon administering it must exercise the highest professional skill and diligence to avoid every possible danger.

"The professional man, no matter how skillful, who leaves an essential link wanting or a danger unguarded in such treatment, is chargeable with negligence, and answerable for any resulting ill consequences."

You will at once notice that the latter charge takes a higher ground of responsibility than the other one quoted, and we can easily determine the relation we, as dentists, in the eye of the law, sustain to the public.

It will now be proper to ascertain what are our relations in case of accidents, and how far a professional man is responsible, whether the injury results from his own acts or from those of an apprentice.

The law on both these points is clearly stated by Chief Justice Tindal, in the case of Hancke vs. Hooper. The facts in this case may be briefly stated as follows: A man came into a surgeon's office, in his absence, and without asking any opinion as to the advisability of performing the operation, requested an apprentice to bleed him in the arm, saying that such treatment had in the past afforded relief to him from a disease from which he had been suffering.

The apprentice bled the patient, and disastrous results followed.

A suit for damages against the surgeon was brought. In his charge to the jury Chief Justice Tindal said:

"The defendant is responsible for the act of his apprentice, therefore, the question is whether you think the injury the plaintiff has sustained is attributable to a want of skill on the part of the young man, or to some accident.

"A surgeon does not become an actual insurer; he is only bound to display sufficient skill and knowledge of his profession. If from some accident, or from some variation in the form of the disease of a particular individual an injury happens, it is not a fault in a medical man.

"It does not appear that the plaintiff consulted the defendant as to the propriety of bleeding him; he took that upon himself and only required the manual operation to be performed.

"The plaintiff must show that the injury was attributable to a want of skill; you are not to infer it. If a person goes into a surgeon's office and asks to be bled, saying that he had found relief from it before, and does not consult the person there as to the propriety of performing the operation, and if there were no indications in the person's appearance that bleeding would be improper, the surgeon would not be liable for the bleeding not effecting the same result as at other times, because it might depend on the constitution of the patient."

In this case the jury found for the surgeon. You will notice that particular stress is laid upon the fact that in the case quoted the patient requested the bleeding to be done, and said he had often obtained relief from such treatment before.

The point I wish to emphasize is this: if a person goes into a dental office and asks for gas to be administered and does not ask advice concerning the advisability of taking it, and should state, to justify the request, that he had often taken it without any ill consequences resulting, one would suppose from the foregoing charge to the jury, that in event of disastrous results the dentist would not be legally responsible.

We all know that there is a wonderful difference in the susceptibility of different individuals, and equal differences in their reparative powers. There is, also, in the same individual at different times, a greater or less power of resistance to disease, and to the lowering of the vitality at different periods of life, and if legally we are not supposed to take these things into account, we are bound by all the moral law to use all necessary precautions, and perform every operation with the same care and careful oversight and attention to details as if we expected each succeeding case to be a variation from the general rule of safety.

In a Pennsylvania case, the patient had previously had a severe fall, which rendered him liable to paralysis, and he was, therefore, not a fit subject for an anæsthetic.

The dentist not knowing this peculiarity, gave an ordinary dose of chloroform, and finding it ineffective gave more, until at length the expected effect was produced and the teeth extracted. But the next day, the patient had an attack of paralysis which his physician attributed to the overdose of chloroform, and he forthwith sued the dentist.

But the court said that a practitioner is not responsible for remote or unknown causes of injury, or for peculiarities of personal history or condition which are not explained to him.

There are many esteemed legal writers who take quite the opposite ground, and say that no physician should ever administer these dangerous agents without first making examination and inquiry into the personal history.

If a physician should do this, why not a dentist? Human life is just as valuable in the dentist's hands as in the surgeon's.

I will now cite another case to show the nature and amount of proof necessary to render a physician liable for negligence or carelessness. The principle from which this may be drawn is amply shown in the case of Rich vs. Pierpont.

Mr. Justice Erle says in his charge to the jury, that "to render a medical man liable for negligence or want of due care or skill, it is not enough that there has been a less degree of care and skill than some other medical man might have shown, or a less degree of care and skill than even he might himself have bestowed; nor is it enough that he himself acknowledges some degree of want of care; there must have been a want of competent and ordinary care and skill, and to such a degree as to have led to a bad result."

Now from these cases it is easily seen that all that can be demanded of a professional man is average, reasonable care and skill, and whenever a question comes up as to whether such skill and care has been exercised, it is a question of fact for a jury.

It will be seen that a professional man is responsible for the deeds of his student or workman, and not responsible for accidents; a point well worth remembering.

The fact of a want of due care and skill must be clearly proved, and cannot be merely inferred.

A professional man is not obliged in law to do his best every time, but must only do ordinarily well. Morally, however, he *is* bound by all that is humane in his nature to use, *every time*, his very best efforts.

The whole drift of the law is to extend to professional men the widest possible leniency consistent with justice, and the sympathies of the court will be with them.

We will now cite another case to show what view the law takes when a professional man of one kind assumes to go into the practice of another.

In an action against a chemist and druggist upon an alleged retainer (as a surgeon and apothecary) to treat the plaintiff for a certain disorder for which mercurial treatment was improper, the charge being negligent treatment, it was held that if the defendant assumed to act as a surgeon or an apothecary he was liable as such, but that these words were not material and might be rejected, the substance of the decision being that he undertook to treat the plaintiff for a disorder and did so negligently or ignorantly; that mercurial treatment in a case for which it was wholly unfit was such negligence or ignorance as would sustain the action.

It is my desire to show by the case just quoted that if we, as dentists, were so unfortunate as to lose a patient when in our chair, under the influence of gas, the question would naturally arise as to our qualifications to administer these agents, and whether we had used all the precautions necessary to insure safety.

The professions of surgery and dentistry were both recognized as distinct callings before the invention of any anæsthetic, and since its discovery nitrous oxide has been adopted by both professions to a greater or less extent, and as its effects and dangers are the same whether administered by a surgeon or dentist, there can be no reason why the members of both professions should not exercise the same precautions in its exhibition, and in the previous examination, or the care of patients during and after its administration. We know that a medical man is not supposed to administer any anæsthetic without knowing by a personal examination the condition of his patient, and if he does administer such an agent without such an examination, and fatal results ensue, he would be amenable to the law. Now, if a physician is liable for the neglect of these precautions, why not a dentist to just the same extent? If we go to Webster for the interpretation of the term

"dentist," we find that he says "a dentist is one who extracts teeth, fills, cleans, and inserts artificial ones;" but he does not say that the administration of anæsthetics comes strictly within the domain of dentistry. If this be true, when a dentist administers gas he is on the domain of the medical man, and he is supposed, in the eye of the law, to take all the precautions for the safety of his patient that the other would have adopted, and the neglect of any of them would place him in the same situation as it would a physician for the same neglect.

When anæsthesia was first invented a surgeon might have been most skillful, and could have amputated a limb with the highest degree of skill and success; yet if he had administered an anæsthetic without a knowledge of its action on the system, and the patient had died from its effects, it would have been no evidence of lack of skill as a surgeon, but simply of his ignorance of the dangers of this agent, and he would undoubtedly have been held legally responsible for the death of his patient. In like manner, a dentist may be one of the most skillful and dextrous operators in either branch, and yet the fact must be patent to all, that if he administer gas and the patient dies from its effects, he would be held as legally responsible for it as if he was ignorant of the first rudiments of operative or mechanical dentistry.

It would not have been of any use for the surgeon in the supposed case stated to have pleaded his knowledge of the human system, or his acknowledged skill as a general surgeon, for the one question which was pertinent was simply whether his knowledge of anæsthetics was such as to justify his administering them. And, gentlemen, if one of us should ever have a death occur during the administration of gas, the law would be silent as to our qualifications for the filling of teeth or inserting artificial ones; the one question by which we would be judged would be, did he understand this agent and its manifestation on the human system, of the action of the heart in normal and abnormal conditions, of the respiratory organs in like conditions, and had he made an examination to see if they were in a condition to make the administration of this agent safe? This, to a certain extent, is not possible for the average dentist to do, and the only line of safety is to demand of each patient that they be accompanied by their family physician, or that they have his written certificate to the effect that it will be safe to administer gas, in which case the only thing you would have to prove in event of death would be that you had administered it with all the usual precautions.

There is one point in relation to anæsthetics which it is always well to bear in mind, and one that can be amply demonstrated, and that is, it is strangely associated with death in the minor operations of surgery.

If we could surround the taking of gas with more restrictions it would aid materially in the preservation of the natural teeth. I believe the experience of all present will justify the conclusion that since gas has come into use and the extraction of teeth has become a painless operation, it has become common for persons to have their teeth extracted by the wholesale, strange and incredible as it seems and have artificial substitutes, rather than undergo the pain and annoyance attending the filling and preservation of them.

I did intend to say something on the question of always having the third person present at each exhibition of gas, for it is well known that strange fancies fill the minds of many persons when under the influence of anæsthetics, but this portion of the subject would prolong this paper to a greater length than would be just to your patience.

The subject is one which could be written on to an indefinite extent, but my object will have been accomplished if I can direct attention to and promote discussion of this, to my mind, the most important question that comes within the range of a dental surgeon, dealing as it does with human life.

